# Non-dispensing pharmacist integrated in the primary care team: effect on the quality of physician’s prescribing, a non-randomised comparative study

**DOI:** 10.1007/s11096-020-01075-4

**Published:** 2020-08-13

**Authors:** Vivianne M. Sloeserwij, Dorien L. M. Zwart, Ankie C. M. Hazen, Judith M. Poldervaart, Anne J. Leendertse, Antoinette A. de Bont, Marcel L. Bouvy, Niek J. de Wit, Han J. de Gier

**Affiliations:** 1 Department of General Practice, Julius Center for Health Sciences and Primary Care, University Medical Center Utrecht (UMCU), Utrecht University, Universiteitsweg 100, 3584 CG Utrecht, The Netherlands; 2 Erasmus School of Health Policy and Management, Erasmus University, Burgemeester Oudlaan 50, 3062 PA Rotterdam, The Netherlands; 3 Department of Pharmaceutical Sciences, Utrecht University, Universiteitsweg 99, 3584 CG Utrecht, The Netherlands; 4 Department of Pharmacotherapy, -Epidemiology and -Economics, University of Groningen, Antonius Deusinglaan 1, Building 3214, 9713 AV Groningen, The Netherlands

**Keywords:** Non-dispensing pharmacist, Pharmaceutical care, Prescribing, Process indicator, Quality

## Abstract

*Background* Especially in elderly with polypharmacy, medication can do harm. Clinical pharmacists integrated in primary care teams might improve quality of pharmaceutical care. *Objective* To assess the effect of non-dispensing clinical pharmacists integrated in primary care teams on general practitioners’ prescribing quality. *Setting* This study was conducted in 25 primary care practices in the Netherlands. *Methods* Non-randomised, controlled, multi-centre, complex intervention study with pre-post comparison. First, we identified potential prescribing quality indicators from the literature and assessed their feasibility, validity, acceptability, reliability and sensitivity to change. Also, an expert panel assessed the indicators’ health impact. Next, using the final set of indicators, we measured the quality of prescribing in practices where non-dispensing pharmacists were integrated in the team (intervention group) compared to usual care (two control groups). Data were extracted anonymously from the healthcare records. Comparisons were made using mixed models correcting for potential confounders. *Main outcome measure* Quality of prescribing, measured with prescribing quality indicators. *Results* Of 388 eligible indicators reported in the literature we selected 8. In addition, two more indicators relevant for Dutch general practice were formulated by an expert panel. Scores on all 10 indicators improved in the intervention group after introduction of the non-dispensing pharmacist. However, when compared to control groups, prescribing quality improved solely on the indicator measuring monitoring of the renal function in patients using antihypertensive medication: relative risk of a monitored renal function in the intervention group compared to usual care: 1.03 (95% CI 1.01–1.05, p-value 0.010) and compared to usual care plus: 1.04 (1.01–1.06, p-value 0.004). *Conclusion* This study did not demonstrate a consistent effect of the introduction of non-dispensing clinical pharmacists in the primary care team on the quality of physician’s prescribing.

This study is part of the POINT-study, which was registered at The Netherlands National Trial Register with trial registration number NTR‐4389.

**Electronic supplementary material:**

The online version of this article (10.1007/s11096-020-01075-4) contains supplementary material, which is available to authorized users.

## Impacts on Practice

- Prescribing indicators might not capture the full effect of non-dispensing pharmacists integrated in primary care teams, when interventions are not specifically targeted upon these indicators.
- A non-dispensing pharmacist integrated in the primary care team improves the monitoring of renal function in patients using diuretics, compared to usual care.
- Future studies on complex, generic interventions should use a mixed methods design to evaluate the effects on quality of care.

## Background

To prevent medication-related harm in the expanding group of elderly with polypharmacy [1, 2], various innovations in the organisation of pharmaceutical care are currently implemented. Integration of clinical pharmacists in primary care teams potentially improves the quality and safety of pharmacotherapy and is currently being evaluated in various formats in Canada, Australia, the United Kingdom and Ireland [3–6]. Also in the Netherlands a *non-dispensing clinical pharmacist *(*NDP*), providing patient-centred pharmaceutical care in close collaboration with the general practitioner (GP), was recently introduced [7].

Clinical pharmacy services provided by such pharmacists in primary care can be either *disease-specific*, tailored to a patient population with a specific medical condition; or *patient-centred*, when provided to a more heterogeneous patient population, such as patients with polypharmacy, patients prescribed at least one medication or patients at risk of medication problems [8].

So far, largest impact of this new care model was found when pharmacists were fully integrated into primary care teams, providing multifaceted interventions and follow up to patients, and with the possibility of face-to-face communication between pharmacist and GP [9, 10]. Effects are mainly found on reducing drug therapy problems and improving proxy outcomes (such as blood pressure control or decreasing HbA1c levels). Yet, effects on prescription quality indicators, commonly used for quality monitoring on practice level by regulators and insurers, is scarce.

### Aim of the study

Despite the promising results, integration of pharmacists in primary care teams has not been adopted widely yet. In this study, we evaluated the effect of patient-centred care delivered by NDPs integrated in primary care teams on medication safety on a practice level. Hereto, we compared NDP-led care with usual care on prescription outcomes, as indicator of quality of pharmaceutical patient care [11].

## Methods

This study was part of the Pharmacotherapy Optimisation through Integration of a Non-dispensing pharmacist in primary care Teams (POINT) study [7]: a non-randomised, controlled intervention study, comparing NDP-led care (intervention group) with two current models of pharmaceutical care (control groups).

The integration of an NDP in primary care teams should be considered as a complex intervention, as it comprises of different interacting components, targets multiple levels of organisation, has variable outcomes and needs to be tailored to the context in which it is implemented [12, 13]. Hence, its evaluation should be multidimensional, including a theoretical framework underlying the expected intervention effect, and assessment of feasibility, effectiveness and related process changes. The theoretical framework as well as results on feasibility and effectiveness have been described elsewhere [14–16]; in the present study we focus on the process changes as measured with indicators that can be derived from computerised healthcare records.

### Ethics approval

The POINT protocol was reviewed by the Medical Ethical Committee of the University Medical Center Utrecht and was deemed not eligible for full assessment (METC protocol number 13-432C). Patient data were extracted anonymously, according to data protection regulations.

### Intervention and control groups

For the POINT study, ten (PharmD) pharmacists were trained as NDPs in a 15-months training program [17]. These NDPs were attached to general practices, collaborating closely with the GPs while being fully integrated in the team. Their key activities were both on a patient level, providing clinical medication reviews and patient consultations for medication problems, as well as on a practice level, educating staff and implementing quality improvement projects. For these quality improvement projects, the NDPs were allowed to select different topics, tailored to the needs of the practice. The NDPs mainly focussed on care for elderly with polypharmacy, but provided pharmaceutical care for younger patients or those with less medications as well (especially in improvement projects). Their role was allowed to evolve during the trial and, if needed, to be adjusted to the needs of daily practice. Most NDPs were relatively at the beginning of their career, with working experience varying from less than 1 year (n = 3), 1–3 years (n = 5) and between 5 and 10 years (n = 2); mainly in community pharmacies (n = 9). The NDPs were blinded for outcome measures (except for the primary outcome: medication-related hospital admissions) during the study period.

Intervention group practices were included only when they were explicitly willing to host an NDP, as willingness of all participating parties to improve pharmaceutical patient care has been recognised as a key condition for successfully implementing an NDP in primary care [3].

Two control groups consisted of the “usual care group”, in which pharmaceutical care was provided by local community pharmacists, and the “usual care plus group”, in which community pharmacists had an additional training [18, 19] in performing clinical medication reviews. Control group practices were matched to the practices in the intervention group as much as possible, with regard to practice size, degree of urbanisation, socioeconomic status and patients’ age distribution. Full details of the design of the POINT-study have been described elsewhere [7].

### Setting and patients

This study was performed in all 25 general practices that participated in the POINT-study. Patients registered in one of these practices, aged 50 years or older and using at least one type of chronic medication (defined as having 3 or more prescriptions per year of the same ATC-3-level medication) were included.

### Study period

We did a pre-post comparison, comparing the prescribing quality during 2013 (pre period) with the prescribing quality in the intervention year, starting June 1st 2014 until May 31st 2015 (post period). The NDPs worked full time in the practices during the intervention year.

### Outcome: quality of prescribing

To evaluate the GPs’ prescribing quality, we used process indicators, as these have been reported most sensitive to differences in quality of care: they are easier to interpret than outcome indicators, and are usually more sensitive to small differences [20].

### Selection of indicators

We collected indicators from literature and policy documents. Indicators were assessed step-wise, including assessment of feasibility, validity, acceptability, reliability and sensitivity to change (Box 1) [11] and health impact. Additional indicators were formulated if needed. For details of the selection procedure, see Online Supplement 1.

**Box 1:** Criteria that quality indicators were assessed on [11]

Criteria	Description
Feasibility	Whether the data needed to calculate the indicator were available in our database
Validity	Whether the content of the indicator was clinically relevant, based upon current guidelines and scientific publications
Acceptability	Whether assessment of the indicator was acceptable for both the patient and the healthcare provider
Reliability	Whether other factors than the prescribing behaviour of the GP could influence the outcome of the indicator, and whether these factors would differ between the study groups
Sensitivity to change	Whether the indicator would detect changes and differences in quality of care

### Data collection

We used anonymised healthcare data routinely extracted from the GPs’ electronic medical records. These data comprised of basic patient characteristics, such as sex and age, and contained all prescribed medications, registered comorbidities and lab tests performed during the study periods. We also collected data on the five months prior to both periods, as for some indicators a timeframe of more than one year was required.

### Sample size calculation

No separate sample size calculation was performed. Data were considered a secondary outcome measurement of the POINT-study, for which a sample size calculation on the primary outcome (medication-related hospitalisations) was performed [7]. Outcomes on the primary outcome have been described elsewhere [16].

### Analysis

Scores on indicators are reported as percentages. Differences in scores over time were reported per study group, but as practices were not randomised, those differences should not be formally compared. Hence, performance per indicator was compared between study groups using mixed models. For a detailed description of the mixed models, see Online Supplement 2. The Consort-checklist for non-randomised trials was used for writing the manuscript (see Online Supplement 3) [21].

## Results

### Indicators of prescribing quality

**Fig. 1:**
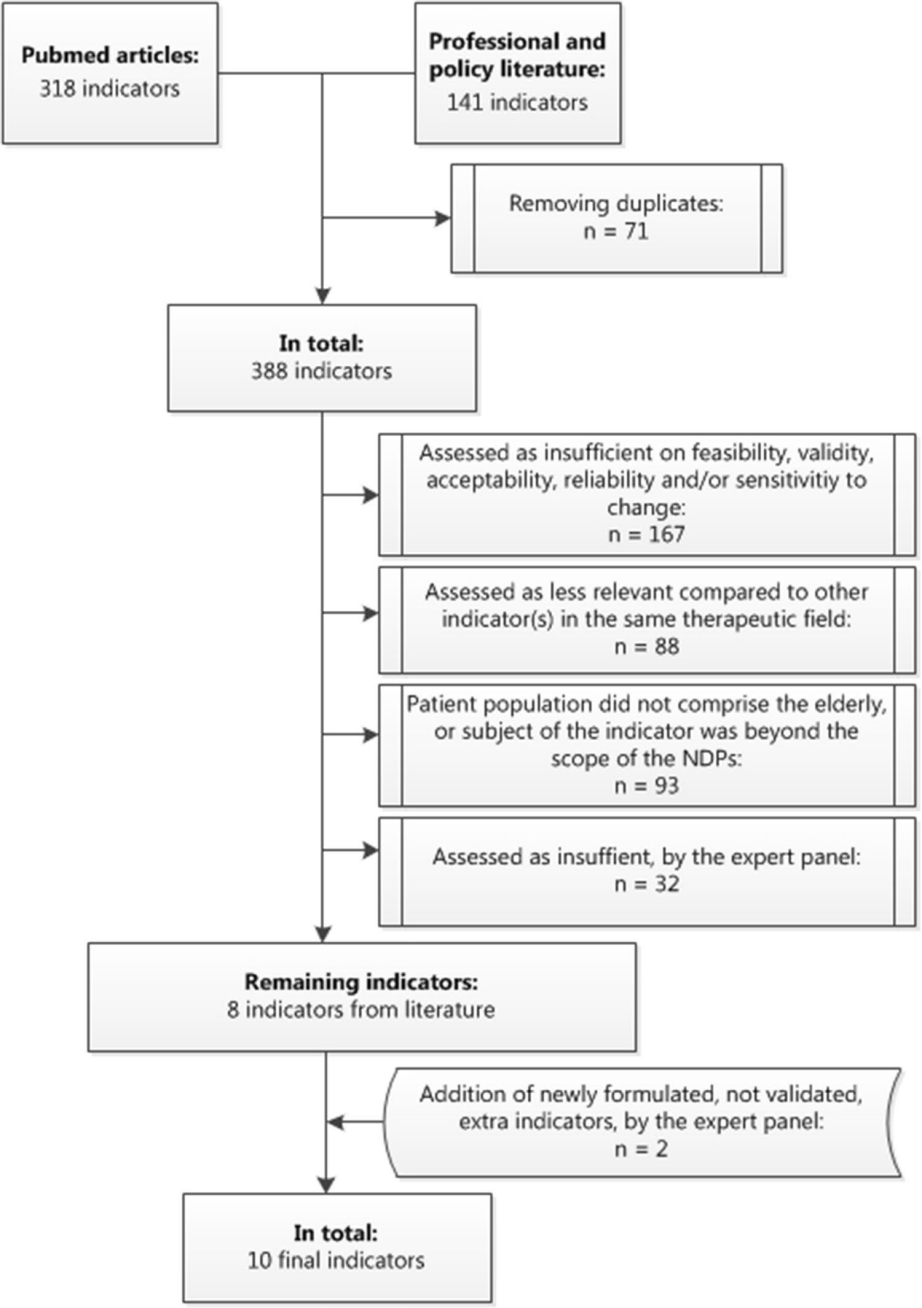
Flowchart of assessment of indicators

The PubMed-search yielded 42 articles, of which 16 were considered relevant. From these, 318 indicators were included. From professional and policy literature we collected an additional 141 indicators. After removing duplicates, 388 indicators remained for assessment, resulting in 8 eligible indicators (see Fig. 1). Of those, two concerned long-term medication use. Because of the nature of our intervention, we needed to alter the definition of ‘long-term’ used in these two indicators in order to enable the indicators to adequately capture change in prescribing quality.

Two additional indicators were formulated by the expert panel. The ten final indicators are summed in Box 2.

**Box 2:** Final set of prescribing quality indicators, per category

**Underprescribing**^a^	
1. PPIs and NSAIDs	Patients aged 70 years or older using non-selective NSAIDs (denominator), using a PPI (numerator)
2. LDL in CVD history	Patients aged younger than 80 years, with a history of cardiovascular disease and at least one measurement of LDL (denominator), having their last LDL-measurement being 2.5 mmol/L or lower with or without statin treatment (numerator)
**Dosing error**^b^	
3. HCT dose	Patients aged 80 years or older using hydrochlorothiazide (denominator), of which the dose is 25 mg/day or higher (numerator)
4. Digoxin dose	Patients aged 70 years or older and using digoxin (denominator), of which the dose is over 0.125 mg/day (if aged 71–85 years) or over 0.0625 mg/day (if aged 86 +) (numerator)
**Therapeutic duplication**^b^	
5. ACEi and ATII-RA	Patients using one or more antihypertensive medications on a chronic basis (denominator), who use both an ACE-inhibitor and an AT-II-antagonist chronically (numerator)
**Contra-indicated**^b^	
6. NSAIDs in CVD history	Patients with a history of cardiovascular disease (denominator), using COX-2 selective NSAIDs (numerator)
**Medication not effective**^b^	
7. Benzodiazepines	Patients aged 65 years or older (denominator), using benzodiazepines for > 300 days per year (numerator)
**Overprescribing**^b^	
8. Antidepressants	All patients (denominator), using antidepressants for > 450 days during period of 17 months (numerator)
**Inadequate monitoring**^a^	
9. Diuretics and renal function	Patients using diuretics and/or RAS-inhibitors (denominator), with known renal function and known potassium levels (numerator)
10. Thyroid medication and function	Patients using thyroid medication (denominator), with known thyroid function (numerator)

*NSAID* Non Steroid Anti-Inflammatory Drug, *PPI* Proton Pump Inhibitor, *LDL* Low Density Lipoprotein, *mg* milligrams, *ACEi* Angiotensin-Converting Enzyme inhibitor, *ATII-RA* Angiotensin II type 2 receptor antagonist, *CVD* Cardiovascular Disease, *COX-2* Cyclo-oxygenase-2, *RAS* Renin-Angiotensin System

Although categories describe potential prescription errors, indicators are formulated as both undesirable care (and hence indeed potential erroneous prescribing) and desirable care (and hence potential correct prescribing):

^a^This category contains indicators representing desirable care, hence a higher score is generally preferable

^b^This category contains indicators representing undesirable care, hence a lower score is generally preferable

All indicators were assessed for the pre and the post period, selecting element of the indicator from that specific study period

‘Using’ was defined as having one or more prescriptions of the medication named

‘Using on a chronic basis’ was defined as having three or more prescriptions of the medication named

Indicators No. 3. and 5. were formulated by the expert panel, and are hence not validated. Indicators No. 7. and 8. contain altered durations of medication use compared to the original indicators, in order to make them susceptible to eventual change

### Participating practices

One NDP stopped during the study period, so we evaluated prescribing quality of 9 practices in the intervention group. In the control groups, for the usual care group 10 practices and for the usual care plus group 6 practices were included. Intervention and control practices were comparable with respect to practice characteristics and patient demographics, except for practice size (see Table 1).

**Table 1:** Baseline characteristics of practices and patient populations

	Intervention (n = 9 practices)	Usual care (n = 10 practices)	Usual care plus (n = 6 practices)
**Practice characteristics**			
Patients aged ≥ 18 years, *median *(*IQR*)	8669 (4765–10,689)	5973 (5371–6646)	6907 (4474 –13,981)
Patients aged ≥ 50 years and using ≥ 1 medication chronically, *median *(*IQR*)	1899 (1262–2301)	1711 (1211–2369)	1768 (1480–3888)
Degree of urbanisation^a^, *mean* ± *SD *(*range*)	1.8 ± 1.1 (1–4)	2.1 ± 0.7 (1–3)	2.2 ± 0.8 (1–3)
Socioeconomic status^b^, *mean* ± *SD *(*range*)	0.9 ± 1.0 (− 1.2–2.2)	0.6 ± 0.9 (− 2.1–1.7)	0.6 ± 0.5 (0–1.2)
Healthcare centre, *n *(%)	7 (78)	7 (70)	3 (50)
Indoor pharmacy^c^, *n *(%)	6 (67)	6 (60)	4 (67)
**Patient characteristics**			
Patients aged ≥ 50 years and using ≥ 1 medication chronically, *n*	15,864	17,609	14,459
Male sex, *n *(%)	7166 (45.2)	7966 (45.2)	6564 (45.4)
Age in years, *median *(*IQR*)	63 (55–72)	63 (55–72)	63 (55–71)
Number of chronic medications per patient, *median *(*IQR*)	3 (2–5)	3 (1–5)	3 (2–5)
Number of comorbidities^d^ per patient, *median *(*IQR*)	2 (1–4)	2 (1–4)	3 (1–4)

*n* number, *IQR* inter quartile range, *SD* standard deviation

^a^Using a five point scale of degree of urbanisation (in which 1 = highly urbanised area, 5 = rural area) [22]

^b^Data from Dutch Social and Cultural Planning Office, using status scores of zip code area of the general practice (in which a higher score represents a higher status) [23]

^c^Being a pharmacy located in the same building as where the general practice is located

^d^Using the UK Quality and Outcomes Framework and overview of chronic diseases developed by the Dutch National Institute for health and Environment [24, 25]

### Fidelity of the intervention

All NDPs implemented quality improvement projects in their practices, but content and scheduling of these projects varied: some projects were implemented right after the NDPs started working in the practice, but others were (partly) implemented only two months before the intervention period ended. This may have limited their effect. The number of projects per practice ranged from 1 to 14 (median 10). Box 3 gives an overview of the covered topics. Six topics matched with clinical themes of the final indicator set.

**Box 3:** Topics of quality improvement projects, implemented by the NDPs (*n*)

	NDPs that implemented the project (n)
**Projects that intervened on specific quality prescribing**	
Underprescribing of PPIs in patients using NSAIDs^a^	6
Underprescribing of inhalation corticosteroids in patients with asthma	5
Underprescribing of statins in patients with a history of cardiovascular disease^a^	4
Underprescribing of calcium and vitamin d in patients using bisphosphonates	4
Underprescribing of vitamin D in patients aged over 70 years	4
Therapeutic duplication of ACEi and AT-II antagonist^a^	6
Contra-indicated NSAIDS in patients with a history of cardiovascular disease^a^	8
Overuse of benzodiazepines^a^	4
Overuse of bisphosphonates	4
Overuse of paracetamol-codeine	1
Overprescribing of antidepressants^a^	1
Overprescribing of alpha-blockers in patients with LUTS	6
Overprescribing of acetylsalicylic acid for primary cardiovascular risk prevention	5
Overprescribing of inhalation corticosteroids in patients with COPD	1
Overprescribing of triptans and starting preventive medication in patients with chronic migraine headache	5
Overprescribing of PPIs	3
Second-line antibiotics	1
First-choice RAS-acting agents in new users	1
**Projects that intervened on comprehensive quality prescribing**	
Medication reconciliation after hospital discharge, taking all used medications into account	5
Compliance with prescribing quality indicators measuring effective prescribing in primary care, defined by the Dutch Institute for Rational Use of Medicine (IVM)	2
**Projects that intervened on organisation of care, underlying quality prescribing**	
Optimise the organisation of referring to fellow GP with additional expertise in a specific (medication) field	1
Optimise the exchange of information on medication prescriptions and medication lists between care providers	2
Optimise registration of contra indications in the medical record	1
Optimise the exchange of information on renal function between GP practice and community pharmacy	1

^a^Topic is represented in the eventual selection of quality prescribing indicators

### Quality of prescribing

In the intervention group, all indicators of desirable prescribing improved, while those measuring undesirable prescribing decreased (Table 2). In the control groups comparable trends were seen, but not for all indicators (for details, see Online Supplement 4).

**Table 2:** Quality indicators of prescribing: percentages per study group and per study period and delta, uncorrected data

Study group	Intervention			Usual care			Usual care plus		
Study period^a^	Pre	Post	Δ	Pre	Post	Δ	Pre	Post	Δ
**Underprescribing**^b^									
1. PPIs and NSAIDs	634/769 (82.4)	596/710 (83.9)	+ 1.5	621/766 (81.1)	619/714 (86.7)	+ 5.6	619/690 (89.7)	551/595 (92.6)	+ 2.9
2. LDL in CVD history	651/1270 (51.3)	798/1416 (56.4)	+ 5.1	757/1307 (57.9)	818/1490 (54.9)	− 3.0	602/979 (61.5)	648/1124 (57.7)	− 3.8
**Dosing error**^c^									
3. HCT dose	127/499 (25.5)	95/453 (21.0)	− 4.5	149/525 (28.4)	124/509 (24.4)	− 4.0	114/372 (30.6)	89/316 (28.2)	− 2.5
4. Digoxin dose	58/175 (33.1)	48/182 (26.4)	− 6.8	47/128 (36.7)	44/150 (29.3)	− 7.4	81/212 (38.2)	57/219 (26.0)	− 12.2
**Therapeutic duplication**^c^									
5. ACEi and ATII-RA	89/5858 (1.5)	77/6336 (1.2)	− 0.3	71/6664 (1.1)	72/7281 (1.0)	− 0.1	131/6223 (2.1)	105/6396 (1.6)	− 0.5
**Contra-indicated**^c^									
6. NSAIDs in CVD history	420/3097 (13.6)	301/3378 (8.9)	− 4.7	378/3398 (11.1)	264/3815 (6.9)	− 4.2	365/2678 (13.6)	202/2893 (7.0)	− 6.6
**Medication not effective**^c^									
7. Benzodiazepines	408/7391 (5.5)	389/7320 (5.3)	− 0.2	401/7750 (5.2)	402/7645 (5.3)	+ 0.1	316/6527 (4.8)	342/6332 (5.4)	+ 0.6
**Overprescribing**^c^									
8. Antidepressants	621/15,864 (3.9)	613/15,935 (3.8)	− 0.1	667/17,609 (3.8)	658/17,693 (3.7)	− 0.1	709/14,459 (4.9)	699/14,283 (4.9)	0.0
**Inadequate monitoring**^b^									
9. Diuretics monitoring	4401/6697 (65.7)	4897/7098 (69.0)	+ 3.3	4735/7384 (64.1)	5079/7815 (65.0)	+ 0.9	4275/6620 (64.6)	4402/6751 (65.2)	+ 0.6
10. Thyroid monitoring	629/968 (65.0)	665/996 (66.8)	+ 1.8	682/1023 (66.7)	721/1084 (66.5)	− 0.2	608/925 (65.7)	661/979 (67.5)	+ 1.8

Indicators are represented as *n numerator/n denominator *(%) for the pre- and post-period, and for the % the difference between both periods is given. No correction for potential confounders was done

*n* number, Δ difference, *PPI* Proton Pump Inhibitor, *NSAID* Non Steroid Anti-Inflammatory Drug, *LDL* Low Density Lipoprotein, *CVD* Cardiovascular Disease, *HCT* Hydrochlorothiazide, *ACEi* Angiotensin-Converting Enzyme inhibitor, *ATII-RA* Angiotensin II type 2 receptor antagonist

^a^Pre-period: the year prior to the intervention year, namely 1 January 2013 until 31 December 2013; Post-period: the year in which the intervention was conducted, namely 1 June 2014 until 31 May 2015

^b^This category contains indicators representing desirable care, hence on average applies: the higher the percentage, the better

^c^This category contains indicators representing undesirable care, hence on average applies: the lower the percentage, the better

After correction for potential confounders and taking the baseline differences into account in mixed models, 4 out of 10 indicators differed between intervention and control group (Table 3, and described in detail in Online Supplement 4).

**Table 3:** Quality indicators of prescribing in the intervention year: comparison between intervention and control groups, corrected for potential confounders (relative risks, 95% CI, p-value)

	Intervention vs. usual care in post-year		Intervention vs. usual care plus in post-year	
**Underprescribing**^a^				
1. PPIs and NSAIDs	0.96 (0.92–1.00)	0.066	**0.91 (0.87–0.94)**	< 0.001
2. LDL in CVD history	1.02 (0.96–1.09)	0.504	0.99 (0.92–1.05)	0.661
**Dosing error**^b^				
3. HCT dose	0.85 (0.60–1.21)	0.373	*0.71 (0.52–0.97)*	0.030
4. Digoxin dose	0.92 (0.65–1.31)	0.652	1.07 (0.67–1.70)	0.780
**Therapeutic duplication**^b^				
5. ACEi and ATII-RA	1.24 (0.88–1.75)	0.223	0.94 (0.58–1.54)	0.808
**Contra-indicated**^b^				
6. NSAIDs in CVD history	**1.27 (1.01**–**1.61)**	0.044	**1.33 (1.05**–**1.69)**	0.019
**Medication not effective**^b^				
7. Benzodiazepines	1.04 (0.78–1.39)	0.797	1.03 (0.77–1.38)	0.849
**Overprescribing**^b^				
8. Antidepressants	1.03 (0.83–1.28)	0.791	0.78 (0.59–1.03)	0.077
**Inadequate monitoring**^a^				
9. Diuretics monitoring	*1.03 (1.01–1.05)*	0.010	*1.04 (1.01–1.06)*	0.004
10. Thyroid monitoring	1.00 (0.94–1.06)	0.873	0.99 (0.93–1.05)	0.697

Differences on scores of indicators are represented as adjusted relative risks with corresponding 95% confidence intervals and p-values. Numbers result from the mixed models, correcting for potential confounders (on patient level: age, sex, the number of medications used and the number of comorbidities; on practice level: socioeconomic status and degree of urbanisation) and if needed, correction for clustering on practice level using random intercepts

^a^Indicator represents desirable care, hence a corrected relative risk greater than 1 resembles a positive intervention effect compared to the control group (in italics if statistically significant), and a relative risk below 1 resembles a negative intervention effect compared to the control group (in bold if statistically significant)

^b^Indicator represents undesirable care, hence a corrected relative risk lower than 1 resembles a positive intervention effect compared to the control group (in italics if statistically significant), and a relative risk greater than 1 resembles a negative intervention effect compared to the control group (in bold if statistically significant)

## Discussion

We assessed the effect of NDPs integrated in primary care teams on the quality of GP prescribing, using 10 selected indicators of prescribing quality. Although the scores of all quality indicators improved in the intervention group, and not in the control groups, we could not demonstrate a consistent favourable effect of NDP introduction on prescribing quality after correction for baseline differences and potential confounders.

### Comparison with existing literature

To our knowledge, only few studies have used process indicators to assess effects of integrating an NDP in primary care teams. In Canada, the effect of integrating a team of a pharmacist and nurse practitioners in primary care was measured using indicators on quality of care for chronic disease management [26]. Most of these indicators concerned prescribing (for example: recommended aspirin in patients with coronary artery disease), but some regarded physical examinations (for example: feet examination in patients with diabetes). Comparable to our study, all indicators improved over time after introduction of the intervention, when examined within the intervention group alone (except for two indicators in which performance was considered relatively high already at baseline). In contrast to our study, the performance of the intervention group was subsequently compared to a control group using a composite indicator. This showed a result in favour of the intervention group. We did not use a composite indicator, as a composite is very dependent on the way it is constructed: differently constructed composite scores can even result into different conclusions being drawn about quality, especially when they include a wide range of medical conditions, different numbers of indicators triggered by a patient and when they include both frequently and more rarely triggered indicators [27].

In a United Kingdom-based study, the effect of a pharmacist-led information technology intervention in primary care on prescribing quality was assessed [28]. In comparison to a control group receiving only simple feedback, significant differences in favour of the intervention group for seven of the 12 measured indicators were found. This result may be explained by the fact that the pharmacist-led information technology intervention was specifically targeted on the measured indicators, while in our study NDP-led care was mainly broadly implemented: focussing on specific interventions can increase the potential to detect change. Although the quality improvement projects implemented by the NDPs were targeted at specific patients groups, the variation in projects among practices was still substantial (see Table 2). Although this variation was explicitly allowed, the resulting heterogeneity and dilution may explain the absence of a consistent effect on the prescribing quality indicators.

### Interpretation of results

We did not find a consistent effect of the integration of NDPs in primary care teams on prescription indicators. Although prescription indicators are considered a suitable measurement for medication safety effects, they may be too specific to assess the true effect of a heterogeneous intervention such as patient-centred NDP-led care.

Still, we found some specific effects that resulted from the NDP intervention: in practices with an integrated NDP, the renal function was monitored more frequently in patients using antihypertensives, compared to in usual care practices. We think this is a result from the clinical medication reviews performed by NDPs, as renal function monitoring was not frequently part of the quality improvement projects. This finding adds to the evidence that the quality of clinical pharmacy services improves when the pharmacist is embedded in clinical practice: NDPs are fully integrated in primary care teams, whilst community pharmacists operate separately from general practice teams. This is also illustrated by a previous finding that recommendations given by NDPs were more frequently followed by GPs, compared to recommendations by community pharmacists [29].

In contrast, we found that in the intervention group patients with a history of cardiovascular disease were prescribed NSAIDs more often as compared to control groups. As almost all NDPs had the use of NSAIDs in CVD patients incorporated in their quality improvement projects (n = 8), this does appear as an unexpected negative outcome. However, we suggest this may be related to the composition of the indicator: whilst quality improvement projects were implemented *during* the intervention year, the indicator measured NSAID-use with a single prescription *at any time* in the intervention year. Hence, it could be that the indicator underestimated the intervention effect, as changes following interventions in patients *after* a first prescription were not captured by the indicator anymore.

### Strengths and limitations

This study has several strengths. We thoroughly assessed a broad selection of indicators, in order to achieve a reliable set to measure the effect of a non-dispensing pharmacist in primary care teams on GPs’ prescribing. Furthermore, the intervention was multifaceted and tailored to the practice and patients’ needs, in a real-world clinical environment. Including patients on a practice level might increase generalisability of results.

Some limitations need to be taken into account as well. First of all, the fact that we—deliberately—chose not to randomise participating practices, may have biased the comparison between the study groups. We corrected for this using mixed models, adjusting for potential relevant baseline characteristics, however bias can’t be fully ruled out.

Second, two limitations concern the use of indicators to measure quality. These limitations are in fact characteristics of indicators that are important to be aware of when interpreting data on indicators, and hence are more a general constraint of using indicators as outcome measurement rather than a specific limitation of this study. First, an indicator can measure only a part of the care provided; it will never reflect the total quality of care. By selecting a *set* of indicators, we tried to gain a wider insight into the quality of prescribing during the provision of NDP-led pharmaceutical care; however, it is still possible that pharmaceutical care improved despite the fact that we couldn’t measure it. Second, evidence based practice requires personalised decisions, sometimes deviating from guidelines. Therefore, optimal prescription outcomes for individual patients may not be optimally reflected in mean indicator scores: “the higher (or the lower) score the better” may not be the aim for every individual [30].

Last, limitations concerning the use of routine healthcare data need to be discussed. First, routine healthcare data are registered for healthcare use, not for research purposes. If data are not registered by GPs, they cannot be measured when using routine healthcare data [31]. Hence, they may reflect quality of registration more than quality of care provided. Second, as data of all patients registered in the practice are extracted, the problem of missing values arises as patients can ‘enter’ the dataset when newly registering and ‘leave’ the dataset when deregistering. Overall, mixed models can handle missing data quite well, but this might still influence our findings. In line with this limitation is the problem of populations changing over time, which changes the case mix of practices. If characteristics of this case mix are related to the indicator, this might influence indicator findings. We tried to exclude such influence as much as possible during the assessment of indicators, however it might still be present to some extent [32]. Another problem of using data of *all* patients registered in the practice is that a final intervention effect might be diluted: in the intervention group, we could not distinguish patients who had received an NDP-led intervention from patients who had no NDP-led intervention. Especially our choice to include a rather broad patient population (aged 50 years and older, using one or more chronic medications) might add to this potential dilution phenomenon. However, as we wanted to measure the complete intervention effect, we preferred this broader patient population over a more detailed population such as patients aged 65 years and older, with polypharmacy), even though the latter may reduce the dilution problem.

So, using extensive data sets such as routinely collected healthcare data is not without limitations. We tried to counter these limitations by applying the same method in each study group and selecting indicators that are least susceptible to misinterpretation. However, we believe interpreting findings based on indicators measured in routine registry data remains uncertain. As a consequence, one should be aware of the above mentioned constraints that might put findings and comparisons at risk.

### Implications for practice

This sub study, focused on measuring the impact of an NDP integrated in primary care with currently used quality indicators, showed no consistent effect of the intervention. Whether this indicates that the NDP does not adequately target the main prescribing problems in GP practice, or that the quality indicators used did not capture the NDP’s effectiveness around these problems remains unsolved. Taking results from the other sub studies of the POINT project into account [16, 29, 33], the latter option may be plausible as our intervention should be considered as complex [13, 34].

## Conclusion

We assessed the effect of NDPs integrated in primary care teams on the quality of prescribing by GPs, using a compiled set of indicators. Although scores on all prescribing quality indicators improved after introduction of the NDP, we could not demonstrate a consistent improvement in prescribing quality in comparison with usual pharmaceutical care. To evaluate such a complex intervention however, in addition to measuring effects on quality, the “how” and “why” of (absence) of effects needs to be addressed as well to fully understand these results.

## Electronic supplementary material

Below is the link to the electronic supplementary material.

Online Supplement 1: Selection of indicators. Supplementary file1 (PDF 124 kb)

Online Supplement 2: Description of the mixed models. Supplementary file2 (PDF 90 kb)

Online Supplement 3: CONSORT 2010 Checklist. Supplementary file3 (PDF 183 kb)

Online Supplement 4: Detailed description of results on quality of prescribing. Supplementary file4 (PDF 93 kb)

## Abbreviations

GP: General practitioner
NDP: Non-dispensing pharmacist
POINT: Pharmacotherapy optimisation through integrating a non-dispensing pharmacist in primary care teams
