# Supplementary material for: Non-dispensing pharmacist integrated in the primary care team: effect on the quality of physician’s prescribing, a non-randomised comparative study
Source: Int J Clin Pharm. 2020 Aug 13;42(5):1293–303. doi: 10.1007/s11096-020-01075-4 (PMC7522101; doi:10.1007/s11096-020-01075-4)
Supplement: Supplementary file 1 — Online Supplement 1: Selection of indicators. Supplementary file1 (PDF 124 kb) [file 11096_2020_1075_MOESM1_ESM.pdf]

Online Supplement 1: Selection of indicators

Literature search

To collect earlier reported indicators, we searched Pubmed and policy documents from government and professional organisations. We searched (mainly Dutch) reports and documents by the Dutch Ministry of Health, Welfare and Sport; the Royal Dutch Pharmacists Association (KNMP); the Dutch College of General Practitioners (NHG); and the Netherlands institute for health services research (NIVEL).

Search strategy:

indicator*[Title] AND prescri*[Title/Abstract] AND (general practice[Title/Abstract] OR primary care[Title/Abstract] OR family practice[Title/Abstract]) AND (full text[sb] AND English[lang]

Assessment of indicators

Duplicate indicators were removed. The remaining set of indicators was assessed step-wise. First, all indicators were evaluated on feasibility, validity, acceptability, reliability and sensitivity to change [11] (Box 1) by two researchers (ZdW and AH). Acceptability was not formally assessed, for all indicators were deemed acceptable as we planned to use routinely collected healthcare data – with no burden or whatsoever to the patient or the healthcare professional. If indicators did not fulfil one or more criteria, they were excluded from further analysis.

Second, indicators meeting all criteria were compared on their clinical themes. In case of overlap, the indicator(s) deemed most clinically relevant was selected. Third, indicators concerning health topics beyond the scope of the NDP in primary care were excluded (for example: cancer treatment).

Finally, the indicators were presented to an expert panel, consisting of two experienced GPs (NdW, DZ) and an experienced community pharmacist (MB). They selected the indicators with the highest health

Corresponding author: Vivianne M Sloeserwij, v.sloeserwij@umcutrecht.nl; Department of General Practice, Julius Center for Health Sciences and Primary Care, University Medical Center Utrecht (UMCU), Utrecht University, The Netherlands.

impact, defined as a combination of a substantial risk of patient harm, and high frequency of occurrence in daily GP practice. In addition, the expert panel checked whether all important aspects of pharmacotherapy were covered. If not, additional indicators were formulated by the expert panel to cover the missing themes, based on evidence and expert opinion. These were not formally validated.

Corresponding author: Vivianne M Sloeserwij, v.sloeserwij@umcutrecht.nl; Department of General Practice, Julius Center for Health Sciences and Primary Care, University Medical Center Utrecht (UMCU), Utrecht University, The Netherlands.
