# Supplementary material for: Non-dispensing pharmacist integrated in the primary care team: effect on the quality of physician’s prescribing, a non-randomised comparative study
Source: Int J Clin Pharm. 2020 Aug 13;42(5):1293–303. doi: 10.1007/s11096-020-01075-4 (PMC7522101; doi:10.1007/s11096-020-01075-4)
Supplement: Supplementary file 2 — Online Supplement 2: Description of the mixed models. Supplementary file2 (PDF 90 kb) [file 11096_2020_1075_MOESM2_ESM.pdf]

Online Supplement 2: Description of the mixed models

Per indicator, we assessed whether patients met the prescribing conditions (dichotomous outcome) in the intervention group compared to the control groups, using an interaction-term between study group and study period. We corrected for several potential confounders, both on the patient level (age, sex, the number of medications and the number of comorbidities), as well as on the practice level (socioeconomic status and degree of urbanisation). Also, we corrected for clustering on practice level using random intercepts, and for repeated patient measurements using random residuals. In case practice clustering-effects were absent, random intercepts were removed. Using a Poisson-distribution, log-link and Robust standard errors, the mixed models resulted in relative risks comparing performances between study groups on each indicator, with 95% confidence intervals.

Analyses were performed with SAS® software, Version 9.4 for Windows, and IBM SPSS Statistics for Windows, Version 23.0 (Armonk, NY).

Corresponding author: Vivianne M Sloeserwij, v.sloeserwij@umcutrecht.nl; Department of General Practice, Julius Center for Health Sciences and Primary Care, University Medical Center Utrecht (UMCU), Utrecht University, The Netherlands.
