# Supplementary material for: Non-dispensing pharmacist integrated in the primary care team: effect on the quality of physician’s prescribing, a non-randomised comparative study
Source: Int J Clin Pharm. 2020 Aug 13;42(5):1293–303. doi: 10.1007/s11096-020-01075-4 (PMC7522101; doi:10.1007/s11096-020-01075-4)
Supplement: Supplementary file 4 — Online Supplement 4: Detailed description of results on quality of prescribing. Supplementary file4 (PDF 93 kb) [file 11096_2020_1075_MOESM4_ESM.pdf]

Online Supplement 4: Detailed description of results on quality of prescribing

Uncorrected data In the intervention group, all indicators of desirable prescribing improved, while those measuring undesirable prescribing decreased (Table 2). In the control groups comparable trends were seen, but not for all indicators. The proportion of cardiovascular disease patients meeting the LDL-target level increased in the intervention group, but decreased in the two control groups. The proportion of patients using antihypertensive drugs that had their renal function checked increased more in the intervention group than in the control groups. Small differences were seen between intervention and control groups in the proportions of patients with benzodiazepines overuse (decrease in the intervention group, increase in both control groups) and PPI-NSAID co-prescription (increased more in both control groups than in the intervention group).

Corrected data After correction for potential confounders and taking the baseline differences into account in mixed models, 4 out of 10 indicators differed between intervention and control group (Table 3). The relative risk of having the renal function checked during use of antihypertensive medication (desired prescribing), was higher in the intervention than in the two control groups (RR 1.03, 95%CI [1.01-1.05] compared to usual care and RR 1.04, 95% CI [1.01 – 1.06] compared to usual care plus). The relative risk of dosing errors of hydrochlorothiazide among elderly (undesired prescribing) was lower in the intervention group than in the usual care plus group (RR 0.71, 95% CI [0.52 – 0.97]), but not different to the usual care group (RR 0.85, 95%CI [0.60 – 1.21]). The relative risk of co-prescription of PPI and NSAID (desired prescribing) was lower in the intervention than in the usual care plus group (RR 0.91, 95% CI [0.87 – 0.94]) but not different from the usual care group (RR 0.96, 95% CI [0.92 – 1.00]). The relative risk of prescribed diclofenac in patients with cardiovascular disease (undesired prescribing) was higher in the intervention group compared to both control groups (RR 1.27, 95% CI [1.01 – 1.61] compared to usual care and RR 1.33, 95% CI [1.05 – 1.69] compared to usual care plus).

Corresponding author: Vivianne M Sloeserwij, v.sloeserwij@umcutrecht.nl; Department of General Practice, Julius Center for Health Sciences and Primary Care, University Medical Center Utrecht (UMCU), Utrecht University, The Netherlands.
